# Advances in Neurological Diseases: Pathogenesis, Diagnosis and Therapeutic Strategies

**DOI:** 10.3390/biomedicines14061355

**Published:** 2026-06-16

**Authors:** Foteini Christidi, Dimitrios Tsiptsios

**Affiliations:** 1 Department of Psychology, School of Philosophy, National and Kapodistrian University of Athens, 15784 Athens, Greece; 2 3rd Department of Neurology, Aristotle University of Thessaloniki, 54124 Thessaloniki, Greece; tsiptsios.dimitrios@yahoo.gr

## 1. Introduction

Neurological diseases are a leading cause of disability and death globally, presenting a diverse and challenging spectrum of conditions that affect millions of individuals [1]. These diseases, encompassing neurodegenerative, cerebrovascular, demyelinating, traumatic, and neurodevelopmental disorders, are frequently characterized by progressive and irreversible decline in motor, sensory, cognitive, or autonomic functions.

Over the past decade, significant progress has been made in deciphering the complex molecular and cellular mechanisms underlying these diseases [2]. However, limitations in early diagnosis, the heterogeneity of clinical presentations, and the lack of disease-modifying treatments underscore the urgent need for translational research that bridges laboratory findings with clinical application.

The goal of this Special Issue was to present novel research findings and state-of-the-art reviews that address these challenges and highlight the current frontiers of neurological disease research.

## 2. Recent Advances in the Field

### 2.1. Understanding Disease Pathogenesis

Our understanding of disease pathogenesis has expanded considerably. Investigations into protein misfolding, mitochondrial dysfunction, neuroinflammation, and excitotoxicity have revealed common threads across diseases like amyotrophic lateral sclerosis (ALS), progressive supranuclear palsy (PSP), and Alzheimer’s disease [3,4].

In this Special Issue, Kleinerova et al. explored the often-overlooked role of sensory dysfunction in ALS and related motor neuron diseases (contribution 1). Their findings indicate that sensory abnormalities are intrinsic to disease pathology, supporting a multisystem model of ALS [5].

Complementing this work, Ciubotaru et al. presented a novel framework integrating fluid biomarkers and brain volumetrics to stratify neurodegenerative subtypes (contribution 2). This multi-modal approach offers a pathway toward precision diagnostics and a deeper understanding of disease heterogeneity [6].

### 2.2. Diagnostic Innovations

Accurate diagnosis—especially in early or atypical presentations—remains a key challenge in neurological care [7,8,9,10,11,12,13]. Several contributions in this Issue tackled this problem directly:

Anyfantakis et al. demonstrated that MRI visual rating scales can reliably distinguish PSP from other forms of Parkinsonism, offering a practical tool for clinicians (contribution 3).

Sandu et al. investigated autonomic nervous system function via heart rate variability metrics in acute ischemic stroke patients, revealing their predictive value for functional recovery and mortality (contribution 4).

In another contribution, Alabdali et al. reviewed the role of stress hyperglycemia as a prognostic biomarker in stroke, emphasizing its potential inclusion in acute care and risk stratification protocols (contribution 5).

### 2.3. Prognosis and Clinical Risk Assessment

The need to reassess assumptions about disease prognosis is also reflected in Hoffmann et al., who investigated the perimesencephalic subarachnoid hemorrhage (PM-SAH) (contribution 6). Although traditionally considered benign, their study suggests PM-SAH may carry longer-term complications than previously recognized [14], advocating for more rigorous post-acute surveillance.

### 2.4. Therapeutic Strategies and Interventions

Moving beyond symptomatic care, therapeutic innovation is increasingly focused on targeting underlying pathology and promoting neuroregeneration [15,16,17]. Chen et al. assess the application of epidural electrical stimulation (EES) in individuals with chronic spinal cord injury, reporting measurable gains in sensory and motor function that may reshape long-term rehabilitative protocols (contribution 7).

Furthermore, Calderone et al. review the role of guided and motor imagery in stroke rehabilitation, underscoring the neuroplastic potential of non-invasive, cognitive-based therapies to restore function (contribution 8).

Nutritional and systemic support also emerge as key elements in patient management. Palma et al. report outcomes of long-term enteral nutrition in neurosurgical patients, emphasizing its value in maintaining metabolic stability and reducing complications during recovery (contribution 9).

## 3. Bridging Gaps: Contributions of the Special Issue

Despite advances, persistent knowledge gaps remain. These include: (a) incomplete characterization of disease heterogeneity, (b) lack of predictive and longitudinal biomarkers, and (c) slow translation of preclinical innovations into patient-centered treatments.

The Special Issue responds to these challenges by offering:

- Redefining mechanisms: by showing how ALS involves sensory systems, and how integrated imaging and biomarkers can classify neurodegenerative subtypes.
- Enhancing diagnostics: through validation of visual rating scales, physiological predictors, and systemic biomarkers.
- Informing prognosis: by revising assumptions about conditions like PM-SAH and emphasizing the prognostic role of autonomic and glycemic measures.
- Advancing therapies: via spinal stimulation, guided motor imagery, and enteral support in complex neurorehabilitation settings.

Together, these contributions reflect a holistic understanding of the neurological disease spectrum, encompassing both central mechanistic research and pragmatic clinical application.

## 4. Future Research Directions

Looking forward, research efforts should converge on the following priorities:

- Multimodal Data Integration: The fusion of genomic, proteomic, imaging, and clinical data—through machine learning and systems biology approaches—will be essential for capturing the multidimensional nature of neurological diseases.
- Precision Medicine Approaches: Stratification of patients based on pathophysiological signatures, rather than symptom clusters alone, will enable the development of tailored combination therapies that simultaneously target multiple pathogenic pathways.
- AI and Digital Biomarkers: Remote monitoring via digital phenotyping and AI-driven analysis of biosignals (e.g., voice, movement, autonomic function) can enable continuous assessment of disease progression and real-time therapeutic adjustment.
- Neurotechnology-Enhanced Rehabilitation: Emerging tools such as brain–computer interfaces, robotic assistive devices, and virtual reality platforms should be rigorously evaluated to optimize post-injury functional recovery.
- Biomarker-Guided Clinical Trials: Adaptive trial designs incorporating early biomarker feedback will enhance drug development efficiency and improve the likelihood of translational success

## 5. Conclusions

This Special Issue of Biomedicines reflects the breadth and innovation of current neurological research. The collected works span fundamental pathogenesis, diagnostic advancements, clinical management, and rehabilitation strategies. Most importantly, they point toward a future in which neurological care is increasingly personalized, data-driven, and integrative.

We thank all contributing authors for their important work and the reviewers for their critical insights. We hope that the ideas and evidence presented in this Issue will serve as a catalyst for future research and clinical innovation.

## References

[1] GBD 2016 Neurology Collaborators (2019). Global, regional, and national burden of neurological disorders, 1990–2016: A systematic analysis for the Global Burden of Disease Study 2016. Lancet Neurol..

[2] Gadhave D.G., Sugandhi V.V., Jha S.K., Nangare S.N., Gupta G., Singh S.K., Dua K., Cho H., Hansbro P.M., Paudel K.R. (2024). Neurodegenerative disorders: Mechanisms of degeneration and therapeutic approaches with their clinical relevance. Ageing Res. Rev..

[3] Fan T., Peng J., Liang H., Chen W., Wang J., Xu R. (2026). Potential common pathogenesis of several neurodegenerative diseases. Neural Regen. Res..

[4] Toader C., Tataru C.P., Munteanu O., Serban M., Covache-Busuioc R.A., Ciurea A.V., Enyedi M. (2024). Decoding Neurodegeneration: A Review of Molecular Mechanisms and Therapeutic Advances in Alzheimer’s, Parkinson’s, and ALS. Int. J. Mol. Sci..

[5] Silani V., Ludolph A., Fornai F. (2017). The emerging picture of ALS: A multisystem, not only a “motor neuron disease. Arch. Ital. Biol..

[6] Valletta M., Briel N., Yuksekel I., Barboure M., Coward A., De Houwer J.F.H., Fawad A., Gonzalez-Mayoral A., Iaccarino G., Martinez-Dubarbie F. (2025). Fluid biomarkers for neurodegenerative diseases: A comprehensive update. Alzheimer’s Res. Ther..

[7] Bede P., Siah W.F., Kassubek J. (2025). Machine-Learning Applications in Frontotemporal Dementia: Challenges, Prospects and Viable Clinical Applications. Eur. J. Neurol..

[8] Sadu V.B., Bagam S., Naved M., Andluru S.K.R., Ramineni K., Alharbi M.G., Sengan S., Khadhar Moideen R. (2025). Optimizing the early diagnosis of neurological disorders through the application of machine learning for predictive analytics in medical imaging. Sci. Rep..

[9] Diogo V.S., Ferreira H.A., Prata D., Alzheimer’s Disease Neuroimaging I. (2022). Early diagnosis of Alzheimer’s disease using machine learning: A multi-diagnostic, generalizable approach. Alzheimer’s Res. Ther..

[10] Tsiara A.A., Plakias S., Kokkotis C., Veneri A., Mina M.A., Tsiakiri A., Kitmeridou S., Christidi F., Gourgoulis E., Doskas T. (2025). Artificial Intelligence in the Diagnosis of Neurological Diseases Using Biomechanical and Gait Analysis Data: A Scopus-Based Bibliometric Analysis. Neurol. Int..

[11] Calikusu F.Z., Akkus S., Kochan Kizilkilic E., Poyraz B.C., Altunc A.T., Kiziltan G., Gunduz A. (2023). Atypical findings: Atypical parkinsonian syndromes or Atypical parkinsonian syndromes look-alikes. Clin. Neurol. Neurosurg..

[12] Mendez M.F. (2006). The accurate diagnosis of early-onset dementia. Int. J. Psychiatry Med..

[13] Vora N., Patel P., Marsool M.D.M., Marsool A.D.M., Sunasra R., Ladani P., Pati S., Khoont D., Prajjwal P., Ranjan R. (2025). Atypical Alzheimer’s dementia: Addressing the subtypes, epidemiology, atypical presentations, diagnostic biomarkers, and treatment updates. Disease-a-Month.

[14] Marquardt G., Niebauer T., Schick U., Lorenz R. (2000). Long term follow up after perimesencephalic subarachnoid haemorrhage. J. Neurol. Neurosurg. Psychiatry.

[15] Li C.P., Wang Y.Y., Zhou C.W., Ding C.Y., Teng P., Nie R., Yang S.G. (2025). Cutting-edge technologies in neural regeneration. Cell Regen..

[16] Dickstein R., Deutsch J.E. (2007). Motor imagery in physical therapist practice. Phys. Ther..

[17] Angeli C.A., Boakye M., Morton R.A., Vogt J., Benton K., Chen Y., Ferreira C.K., Harkema S.J. (2018). Recovery of Over-Ground Walking after Chronic Motor Complete Spinal Cord Injury. N. Engl. J. Med..

